# Adverse drug reaction assessment of pembrolizumab in cervical cancer treatment: a real-world pharmacovigilance study using the FAERS database

**DOI:** 10.3389/fimmu.2025.1582050

**Published:** 2025-04-08

**Authors:** Huiping Zhang, Zhuo Zhou, Juan Wang, Shan Wang, Jie Ren, Ming Zhang, Mingyi Yang

**Affiliations:** ^1^ Department of Obstetrics and Gynecology, Northwest University First Hospital, Xi’an, Shaanxi, China; ^2^ Department of General Practice, Honghui Hospital, Xi’an Jiaotong University, Xi’an, Shaanxi, China; ^3^ Department of Joint Surgery, Honghui Hospital, Xi’an Jiaotong University, Xi’an, Shaanxi, China

**Keywords:** pembrolizumab, cervical cancer, immunotherapy, immune checkpoint inhibitors, immune-related adverse events

## Abstract

**Objective:**

Advanced cervical cancer remains associated with high mortality rates. While pembrolizumab has improved clinical outcomes in cervical cancer, the therapeutic efficacy in advanced stages is often compromised by immune-related adverse events (irAEs). This study aimed to systematically analyze pembrolizumab-associated adverse events (AEs) in cervical cancer using the FDA Adverse Event Reporting System (FAERS) database, providing new insights for optimizing clinical practice.

**Methods:**

AE reports related to pembrolizumab in cervical cancer were extracted from the FAERS database (Q1 2016 to Q4 2024). Disproportionality analyses were performed using multiple algorithms, including the reporting odds ratio (ROR), proportional reporting ratio (PRR), Bayesian confidence propagation neural network (BCPNN), and multi-item gamma Poisson shrinker (MGPS). AEs were classified by system organ class (SOC) and preferred term (PT) based on the Medical Dictionary for Regulatory Activities (MedDRA), then ranked by frequency and signal strength.

**Results:**

A total of 646 pembrolizumab-related AE reports in cervical cancer were identified. Age distribution peaked at 45–65 years cohort (32.75%), followed by 18–44 years (12.85%), 66–75 years (11.76%), and >75 years (4.64%). Among 270 AE reports with documented onset timelines, events predominantly occurred 3–6 months after pembrolizumab initiation (n=114, 41.36%). Clinical outcomes were categorized as other (52.80%), hospitalization (27.00%), death (10.25%), unknown (6.06%), life-threatening (2.77%), and disability (1.12%). Predominant AEs involved hematologic, endocrine, dermatologic, neurologic, gastrointestinal, urinary, and reproductive systems.

**Conclusion:**

This real-world pharmacovigilance study systematically characterizes pembrolizumab-associated AEs in cervical cancer, identifying high-signal events such as hematologic disorders, endocrine dysfunction, and dermatologic toxicities. These findings provide critical evidence for risk stratification and safety monitoring in clinical practice, emphasizing the need for organ-specific vigilance during the 3–6 months treatment window.

## 1 Introduction

Cervical cancer, a prevalent gynecologic malignancy among perimenopausal women globally, exhibits marked regional heterogeneity in disease burden. According to 2021 global cancer statistics, approximately 670,000 new cases and 296,667 deaths were attributed to cervical cancer, with over 85% of cases concentrated in low- and middle-income countries (1). Despite the widespread HPV vaccination and early screening that have effectively reduced the incidence in high-income regions, the age-standardized mortality rate in low-income regions continues to rise at an annual rate of 1.7%, leading to a growing global disease burden due to uneven distribution of healthcare resources (2). Traditional treatment options for cervical cancer include surgery, radiotherapy, and chemotherapy. However, with the advancement of molecular biology, the application of anti-angiogenic drugs, such as bevacizumab, has improved the prognosis of cervical cancer to some extent (3). Nevertheless, approximately 15%-20% of patients with locally advanced disease and 60% of metastatic patients experience poor prognosis due to primary resistance or relapse after treatment, with a 5-year survival rate of less than 20% (4), highlighting the urgent need for novel therapeutic strategies.

With the deepening research into the tumor microenvironment (TME), the mechanisms of molecules such as CTLA-4, PD-1, and PD-L1 have been gradually elucidated. These molecules regulate immune cell functions, promoting immune escape of tumor cells and providing favorable conditions for tumor growth and metastasis (5). Based on this, immune checkpoint inhibitors (ICIs) have been progressively incorporated into the treatment regimens for malignant tumors. These currently cover melanoma, non-small cell lung cancer (NSCLC), hematologic malignancies (such as classical Hodgkin lymphoma), and gynecologic malignancies (ovarian cancer, cervical cancer), with the application of ICIs significantly improving the survival outcomes of these cancer patients (6). ICIs primarily target CTLA-4, PD-1, or PD-L1, with CTLA-4 inhibitors acting during the priming phase of immune activation, while PD-1/PD-L1 inhibitors disrupt effector-phase immune regulation (7). Pembrolizumab is one of the most widely used immune checkpoint inhibitors in cervical cancer. It activates the immune system by blocking the binding of PD-1 to its ligand, thereby promoting immune cells to recognize and attack cancer cells (8). After being approved by the he U.S. Food and Drug Administration (FDA) for the treatment of melanoma in 2014, the prognosis of patients was significantly improved. Subsequently, pembrolizumab has been applied in clinical settings for various tumors, including NSCLC, urothelial carcinoma, head and neck cancer, liver cancer, and renal cell carcinoma, all with favorable outcomes (9). In 2021, pembrolizumab was FDA-approved for use in patients with chemotherapy-resistant, recurrent, or PD-L1-high expressing cervical cancer (10). In a KEYNOTE-826 trial involving 617 cervical cancer cases, the Pembrolizumab plus chemotherapy group showed significantly improved progression-free survival and overall survival compared to the chemotherapy-only group (11). Furthermore, multiple studies have similarly demonstrated that pembrolizumab combined with chemotherapy significantly improved patient prognosis in the treatment of locally advanced cervical cancer (12, 13).

Despite the significant potential of pembrolizumab in improving the prognosis of cervical cancer, its efficacy is somewhat limited by various immune-related toxicities, collectively known as immune-related adverse events (irAEs) (14). The immune responses activated by ICIs can affect all organs in the body, with the most commonly involved organs being the skin, liver, gastrointestinal tract, and endocrine glands (15, 16). The incidence and severity of irAEs are closely related to the ICI target, with grade 3-4 irAEs occurring at significantly higher rates with anti-CTLA-4 monoclonal antibodies (such as ipilimumab) than with anti-PD-1 and anti-PD-L1 monoclonal antibodies. PD-1 inhibitor-related irAEs predominantly involve skin toxicity (maculopapular rash, vitiligo), thyroid dysfunction, and pneumonia (17). Current evidence on ICI treatment for cervical cancer largely focuses on efficacy evaluation, while studies on irAEs specific to this patient population remain significantly lacking. A systematic review of ICIs in gynecological oncology indicated that only 12% of clinical trials reported detailed irAE profiles for the cervical cancer subgroup (18), and there is a lack of toxicity management models based on real-world data. Based on this, our study innovatively utilizes the FAERS database to systematically explore signals of pembrolizumab in the treatment of cervical cancer, especially irAEs. These findings provide important data to support the development of personalized pembrolizumab monitoring strategies in the clinical management of cervical cancer.

## 2 Methods

### 2.1 Data acquisition

The raw data used for data mining was obtained from the FDA’s official website via the FAERS database. The database offers data in two formats (ASCII and XML packages) for download. In this study, we downloaded the raw ASCII data package from the first quarter of 2016 to the Fourth quarter of 2024 for statistical analysis.

### 2.2 Data deduplication

The FDA’s official guidance documents provide rules for data deduplication and a list of reports that need to be excluded. This study strictly followed the FDA’s official guidelines for data cleaning. The deduplication process was carried out in two steps: First, using the FDA’s recommended method for removing duplicate reports, we selected the PRIMARYID, CASEID, and FDA_DT fields from the DEMO table. The reports with the same CASEID were sorted by CASEID, FDA_DT, and PRIMARYID, and the report with the largest FDA_DT value was retained. In cases where both CASEID and FDA_DT were the same, the report with the largest PRIMARYID value was kept. Secondly, starting from the first quarter of 2016, each quarterly data package included a list of reports to be deleted. After deduplication, the reports were excluded based on the CASEID listed in the delete report list. An example of the deduplication report is shown in **Table 1**.

**Table 1:** Deduplication report example.

Primaryid	Caseid	FDA_DT	Operation
4271842	3070600	20060106	Delete
4271943	3070600	20060106	Delete
4283856	3070600	20060129	Delete
4314676	3070600	20060308	Keep

### 2.3 Application of the MedDRA dictionary

The latest version of the MedDRA dictionary is used to revise the preferred term (PT) names in the FAERS database, and to obtain the system organ class (SOC) and preferred terms (PT) from the latest MedDRA dictionary for subsequent analysis.

### 2.4 Handling of false positive adverse events

The database sets the role cod (reporting role code of the drug in the event) in the DRUG table to identify true “drug-adverse event” signals. The role cod is divided into four categories: PS (primary suspected drug), SS (secondary suspected drug), C (concomitant), and I (interaction). In this study, we identified cases in the DRUG file using “Pembrolizumab”, and selected role cod as PS to improve accuracy. Additionally, to reduce the false positive rate, we applied the imbalance ratio measurement method to mine true “drug-adverse event” signals.

### 2.5 Statistical methods

This study utilized R 4.4.2 for statistical analysis, while multivariable logistic regression was conducted using SPSS 29.0. In pharmacovigilance research, we applied disproportionality analysis to compare the proportion of specific adverse reactions related to one or more drugs with the proportion of adverse reactions for the same drug reported in the entire database. Based on disproportionality analysis, we also used the imbalance ratio measurement method to identify associations between drugs and AEs. The four main specific indicators used to assess drug-related AE signals are the reporting odds ratio (ROR), proportional reporting ratio (PRR), Bayesian confidence propagation neural network (BCPNN), and multi-item gamma Poisson shrinker (MGPS) algorithms. These methods are based on a 2x2 contingency table for statistical analysis by calculating the relative frequency of target adverse reactions caused by target drugs in the database over a period of time, thereby evaluating the statistical relationship between a specific drug and a particular AE. As shown in the table below, ‘a’ refers to the number of reports containing both the target drug and target drug adverse reactions; ‘b’ refers to the number of reports containing other drug adverse reactions with the target drug; ‘c’ refers to the number of reports containing target drug adverse reactions with other drugs; and ‘d’ refers to the number of reports containing both other drugs and other drug adverse reactions. The imbalance ratio measurement method 2x2 contingency table is shown in **Table 2**.

**Table 2:** Imbalance ratio measurement method 2x2 contingency table.

Types of Drugs	Target Adverse Event Reports	Other Adverse Event Reports	Total
Target drug	a	b	a+b
Other drugs	c	d	c+d
Total	a+c	b+d	a+b+c+d

The ROR is a statistical indicator used in pharmacovigilance to assess the association between a specific adverse event and a specific drug, and compare it with all other drugs in the database (19). The conditions for generating an ROR signal are: 95% CI (lower bound) ≥ 1, a ≥ 3. The formula for calculating ROR is as follows:


$$ROR=\frac{ad}{bc}$$

$$SE(\ln ROR)=\sqrt{\left(\frac{1}{a}+\frac{1}{b}+\frac{1}{c}+\frac{1}{d}\right)}$$

$$95\%CI=e^{\ln(ROR)\pm \sqrt[{1.96}]{\left(\frac{1}{a}+\frac{1}{b}+\frac{1}{c}+\frac{1}{d}\right)}}$$

The PRR is one of the methods for performing quantitative analysis of spontaneous reporting systems. It determines the incidence rate of a specific adverse drug event (ADE) associated with exposure to a particular drug by analyzing the ratio of the ADE in those exposed to the drug compared to the ratio of ADEs occurring without exposure to the drug (20). The conditions for generating a PRR signal are: a ≥ 3, 95% CI (lower bound) ≥ 1, and PRR ≥ 2. The formula for calculating PRR is as follows:


$$PRR=\frac{a/(a+b)}{c/(c+d)}$$

$$SE(\text{ln }PRR)=\sqrt{\left(\frac{1}{a}-\frac{1}{a+b}+\frac{1}{c}-\frac{1}{c+d}\right)}$$

$$95\%CI=e^{\ln(PRR)\pm \sqrt[{1.96}]{\left(\frac{1}{a}-\frac{1}{a+b}+\frac{1}{c}-\frac{1}{c+d}\right)}}$$

The Multi-item Hypergeometric Reporting Algorithm (MHRA) builds upon the PRR by also considering the χ² value and the number of reports. Compared to ROR and PRR, it is more rigorous. A signal is generated when PRR &gt; 2, χ² ≥ 4, and the number of reports (a) ≥ 3 are all satisfied simultaneously. The conditions for generating a signal using the MHRA algorithm are: a ≥ 3, PRR &gt; 2, and χ² ≥ 4. The formula for calculating MHRA is as follows:


$$PRR=\frac{a/(a+b)}{c/(c+d)}$$

χ2=[(ad−bc)^2](a+b+c+d)/[(a+b)(c+d)(a+c)(b+d)]

As a probabilistic uncertain reasoning method, the BCPNN serves as an important tool for handling uncertain information (21). Typically, the BCPNN determines potential associations between drugs and adverse reactions by calculating the Information Component (IC). The signal generation criterion for the BCPNN algorithm is defined as: IC - 2SD > 0. The computational formulas are as follows:


$$\begin{array}{l} IC=\log_{2}\frac{a(a+b+c+d)}{\left(a+c)(a+b\right)}\\ E(IC)=\log_{2}\frac{\left(a+\gamma 11)(a+b+c+d+\alpha )(a+b+c+d+\beta \right)}{\left(a+b+c+d+\gamma )(a+b+\alpha 1)(a+c+\beta 1\right)}\\ V(IC)=\frac{1}{{\left(\ln2\right)}^{2}}\{[\frac{(a+b+c+d)-a+\gamma +\gamma 11}{\left(a+\gamma 11)(1+a+b+c+d+\gamma \right)}]+[\frac{(a+b+c+d)-(a+b)+\alpha -\alpha 1}{\left(a+b+\alpha 1)(1+a+b+c+d+\alpha \right)}]+[\frac{(a+b+c+d)-(a+c)+\beta -\beta 1}{\left(a+c+\beta 1)(1+a+b+c+d+\beta \right)}]\} \end{array}$$

$$\begin{array}{l} \gamma =\gamma 11\frac{\left(a+b+c+d+\alpha )(a+b+c+d+\beta \right)}{\left(a+b+\alpha 1)(a+c+\beta 1\right)}\\ \text{IC-2SD= E(IC) -2V(IC)\textasciicircum{}0}.5 \end{array}$$

The MGPS method is currently adopted by FDA. Its core lies in the calculation of the Empirical Bayes Geometric Mean (EBGM) (22). Typically, the MGPS algorithm identifies potential drug-adverse reaction signals based on the criterion EB05 ≥ 2, where EB05 represents the lower limit of the 95% confidence interval for EBGM.


$$\begin{array}{l} EBGM=a(a+b+c+d)/(a+c)/(a+b)\\ 95\%CI=e^{\ln(EBGM)\pm \sqrt[{1.96}]{\left(\frac{1}{a}+\frac{1}{b}+\frac{1}{c}+\frac{1}{d}\right)}} \end{array}$$

To investigate factors associated with immune-induced adverse events, we further performed multivariable logistic regression analysis. The independent variables included age, weight and off-label use. Age was categorized into two groups: <65 years and ≥65 years, while weight was divided into three categories: <50 kg, 50–100 kg, and >100 kg. P-values less than 0.05 were considered statistically significant.

## 3 Results

### 3.1 Data acquisition results

**Figure 1:**
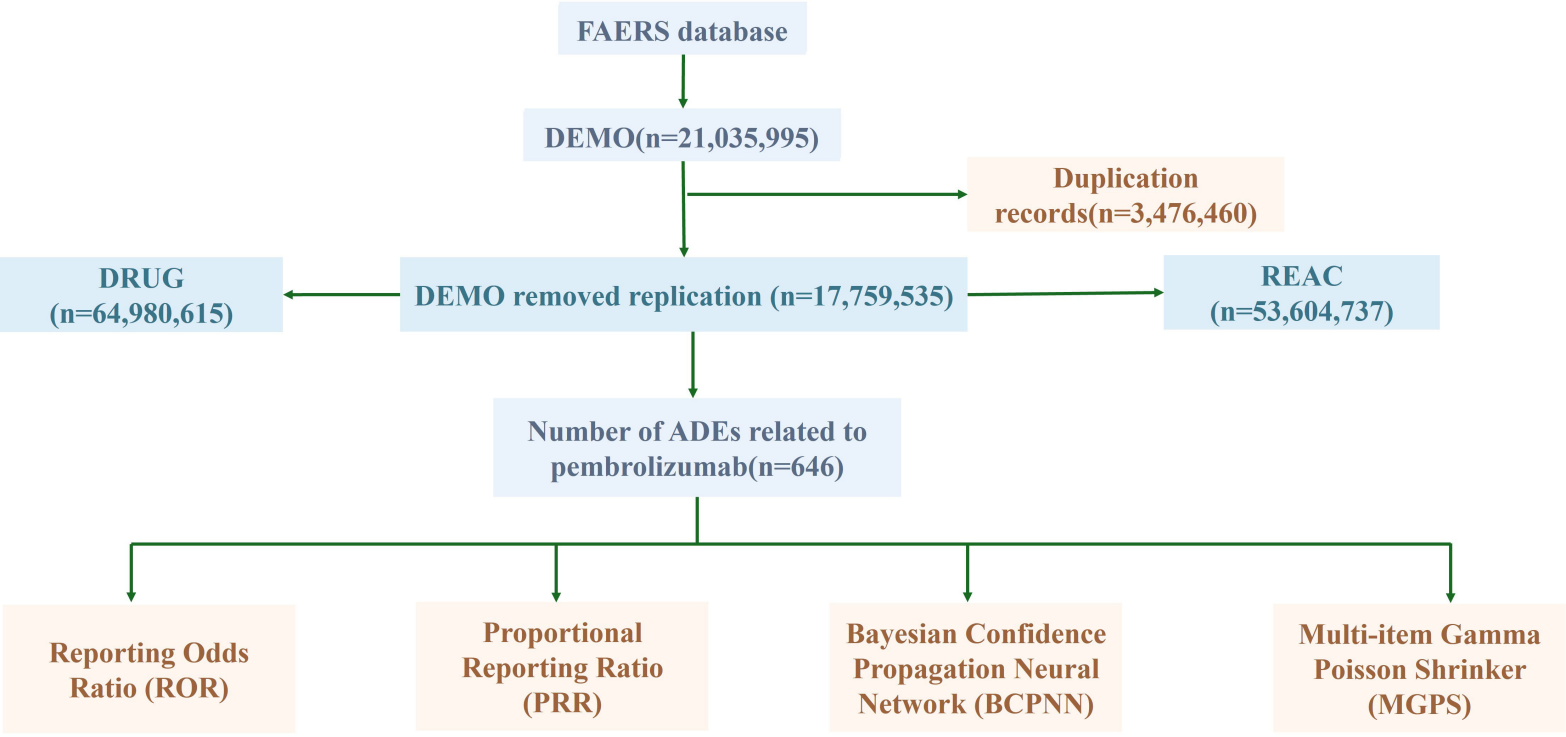
The flow diagram of screening reports containing pembrolizumab from the FAERS database.

From the first quarter of 2016 to the fourth quarter of 2024, a total of 890 adverse event (AE) reports associated with pembrolizumab in cervical cancer were retrieved from the FAERS database. After stepwise deduplication and rigorous quality control, 646 unique AE reports were ultimately included for analysis (**Figure 1**).

### 3.2 Basic information of AE reports

**Figure 2:**
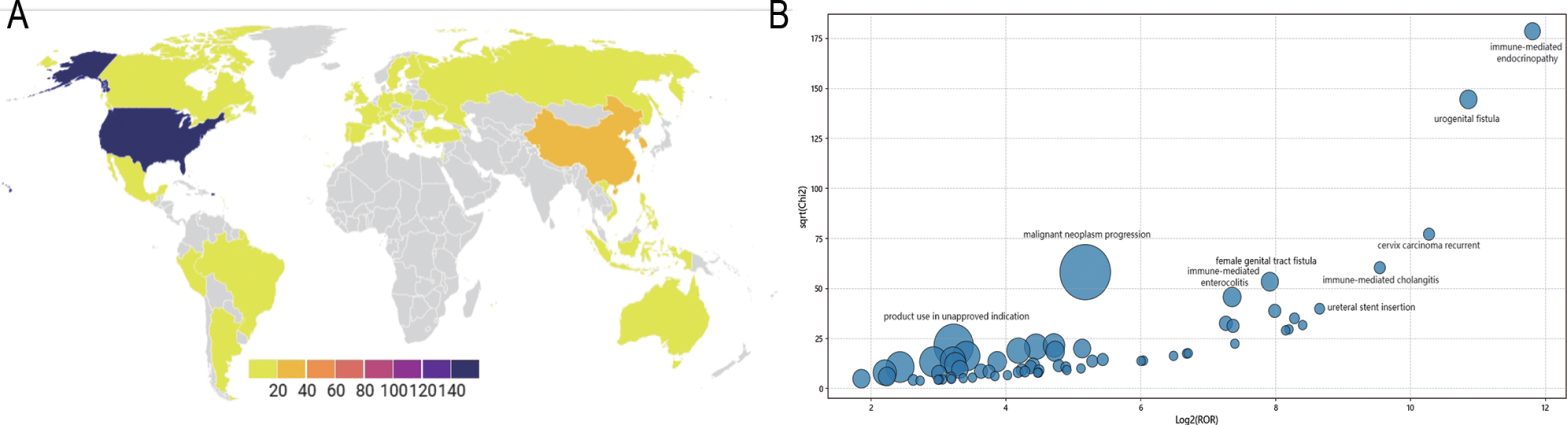
Pembrolizumab-related AEs reporting and distribution **(A)** Pembrolizumab-related AEs reporting distribution map across different countries; **(B)** Pembrolizumab-related AEs distribution bubble chart.

**Figure 3:**
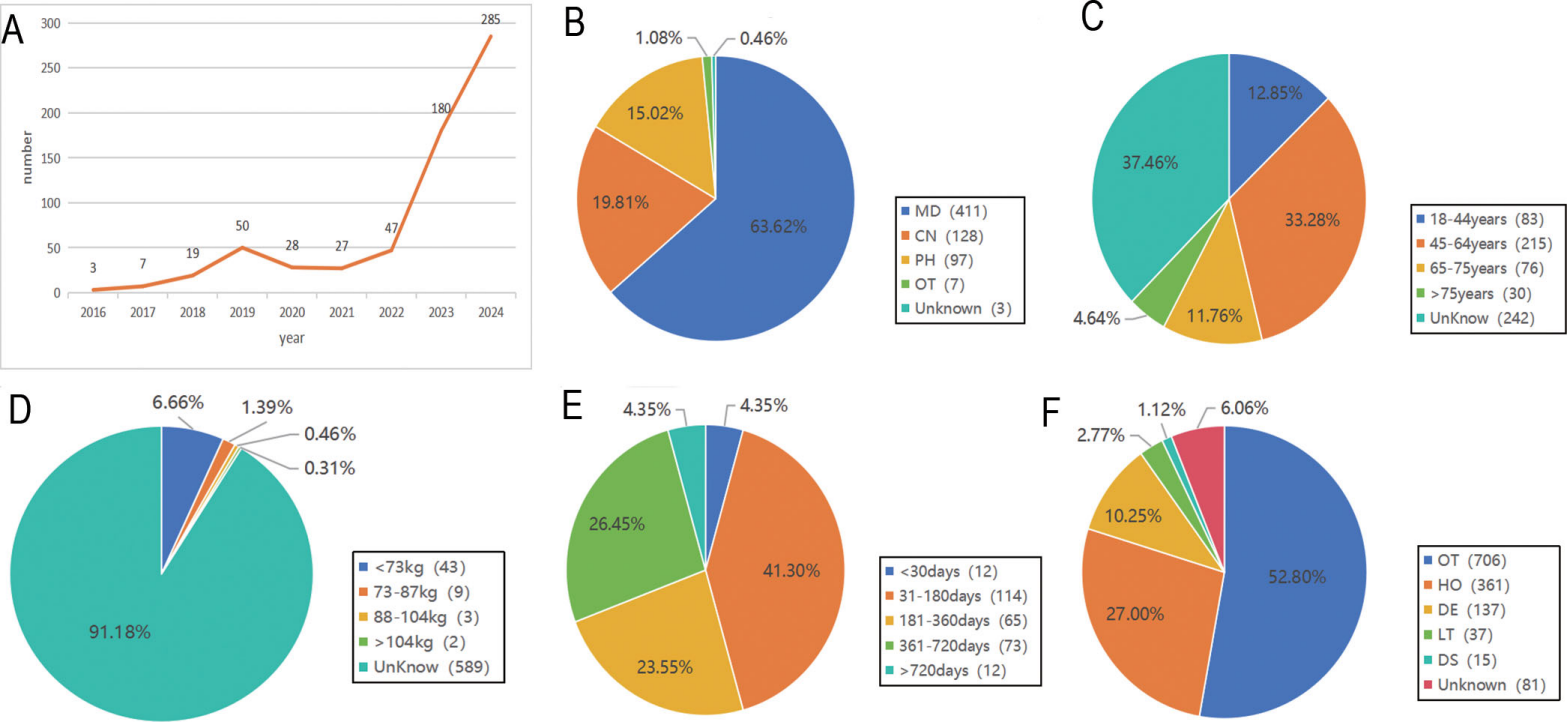
General information of Pembrolizumab-related AEs reports. **(A)** The Annual Reporting Line Chart of Pembrolizumab-Related AEs; **(B)** The Distribution of Reporters’ Identities for Pembrolizumab-Related AEs; **(C)** The Age Distribution of Reported Pembrolizumab-Related AEs; **(D)** The Weight Distribution of Reported Pembrolizumab-Related AEs; **(E)** The Onset Time Distribution of Pembrolizumab-Related AEs; **(F)** The Outcome Distribution of Pembrolizumab-Related AEs.

AE reports were submitted from 35 countries, with Japan (53.56%) contributing the highest proportion, followed by the United States (23.53%), South Korea (5.88%), China (3.09%), France (2.01%), and others. Pembrolizumab-associated AEs in cervical cancer primarily involved product use in unapproved indication, malignant neoplasm progression, and immune-mediated cholangitis (as shown in **Figure 2**). Overall, AE reporting frequency demonstrated a year-over-year increase. Physicians submitted the majority of reports (63.62%), followed by nurses (19.81%) and other healthcare professionals (15.02%). Age distribution revealed the highest AE incidence in the 45–65 years cohort (32.75%), followed by 18–44 years (12.85%), 66–75 years (11.76%), and >75 years (4.64%). Weight distribution showed <73 kg (6.66%) as the most prevalent category, followed by 73–87 kg (1.39%) and 88–104 kg (0.46%). Among 270 AE reports with documented onset timelines, events predominantly occurred 3–6 months after pembrolizumab initiation (n=114, 41.36%). Clinical outcomes associated with pembrolizumab use in cervical cancer were categorized as other (52.80%), hospitalization (27.00%), death (10.25%), unknown (6.06%), life-threatening (2.77%), and disability (1.12%) (as shown in **Figure 3**).

### 3.3 Frequency distribution of AE signals

The top 30 most frequently reported AEs included the following: Here’s the organized list of the provided terms: Malignant Neoplasm Progression; Product Use in Unapproved Indication; Anemia; Neutropenia; Adverse Event; Neuropathy Peripheral; Inappropriate Schedule of Product Administration; Skin Disorder; Neutrophil Count Decreased; Myelosuppression; Febrile Neutropenia; Hypothyroidism; Drug Eruption; Immune-mediated Enterocolitis; Female Genital Tract Fistula; Renal Impairment; Intestinal Perforation; Platelet Count Decreased; Urogenital Fistula; Colitis; Immune-mediated Endocrinopathy; Hepatic Function Abnormal; Transfusion; Proteinuria; Immune-mediated Hepatic Disorder; Immune-mediated Hypothyroidism; Erythema Multiforme; Eastern Cooperative Oncology Group Performance Status Worsened; Cytokine Release Syndrome; Cervix Carcinoma Recurrent.(as shown in **Table 3**).

**Table 3:** Top 30 signal frequencies of AEs at the SOC level for pembrolizumab in the treatment of cervical cancer.

Number	PT	N	ROR (95%CI)	PRR (X^2^)	IC025/IC-2SD	EBGM05
1	malignant neoplasm progression	104	36.25 (29.75,44.18)	34.33 (3367.13)	4.41	28.14
2	product use in unapproved indication	63	9.38 (7.29,12.05)	9.1 (455.83)	2.65	7.08
3	anemia	32	5.4 (3.81,7.66)	5.33 (112.79)	1.73	3.76
4	neutropenia	31	7.61 (5.34,10.86)	7.51 (175.17)	2.13	5.26
5	adverse event	30	10.68 (7.45,15.32)	10.53 (259.05)	2.49	7.34
6	neuropathy peripheral	26	9.3 (6.32,13.7)	9.19 (190.0)	2.26	6.24
7	inappropriate schedule of product administration	22	4.62 (3.03,7.03)	4.58 (61.66)	1.38	3.01
8	skin disorder	22	21.86 (14.35,33.28)	21.62 (432.49)	2.9	14.19
9	neutrophil count decreased	22	18.24 (11.98,27.77)	18.04 (354.05)	2.77	11.84
10	myelosuppression	19	26.28 (16.72,41.31)	26.03 (457.12)	2.88	16.55
11	febrile neutropenia	19	9.54 (6.07,14.99)	9.45 (143.74)	2.08	6.01
12	hypothyroidism	14	14.67 (8.67,24.81)	14.57 (176.88)	2.19	8.6
13	drug eruption	14	26.61 (15.72,45.02)	26.42 (342.18)	2.54	15.6
14	immune-mediated enterocolitis	13	164.0 (94.91,283.4)	162.9 (2080.12)	2.92	93.74
15	female genital tract fistula	12	241.76 (136.73,427.47)	240.25 (2835.64)	2.82	134.77
16	renal impairment	12	4.72 (2.68,8.33)	4.7 (34.99)	1.07	2.66
17	intestinal perforation	12	35.12 (19.9,61.97)	34.9 (394.79)	2.47	19.76
18	platelet count decreased	12	3.63 (2.06,6.41)	3.62 (22.74)	0.79	2.05
19	urogenital fistula	12	1860.84 (1036.36,3341.22)	1849.18 (20836.24)	2.86	968.11
20	colitis	11	9.99 (5.52,18.07)	9.94 (88.44)	1.67	5.49
21	immune-mediated endocrinopathy	10	3595.38 (1861.09,6945.79)	3576.6 (31817.91)	2.53	1647.98
22	hepatic function abnormal	9	8.06 (4.19,15.52)	8.03 (55.39)	1.32	4.17
23	transfusion	7	21.19 (10.09,44.53)	21.12 (134.1)	1.56	10.05
24	proteinuria	7	12.42 (5.91,26.09)	12.38 (73.2)	1.33	5.89
25	immune-mediated hepatic disorder	7	153.84 (73.1,323.77)	153.28 (1053.48)	1.91	72.45
26	immune-mediated hypothyroidism	6	254.18 (113.65,568.47)	253.39 (1495.28)	1.68	112.32
27	erythema multiforme	6	20.66 (9.27,46.06)	20.6 (111.83)	1.35	9.23
28	eastern cooperative oncology group performance status worsened	6	165.59 (74.13,369.89)	165.07 (972.95)	1.66	73.48
29	cytokine release syndrome	6	13.49 (6.05,30.06)	13.45 (69.11)	1.18	6.03
30	cervix carcinoma recurrent	5	1241.29 (506.6,3041.5)	1238.05 (5926.99)	1.37	484.58

PT, Preferred Terms; ROR, Reporting Odds Ratio; PRR, Proportional Reporting Ratio; IC025/IC-2SD, nformation Component Lower Limit; EBGM05, Empirical Bayes Geometric Mean 5th Percentile.

**Figure 4:**
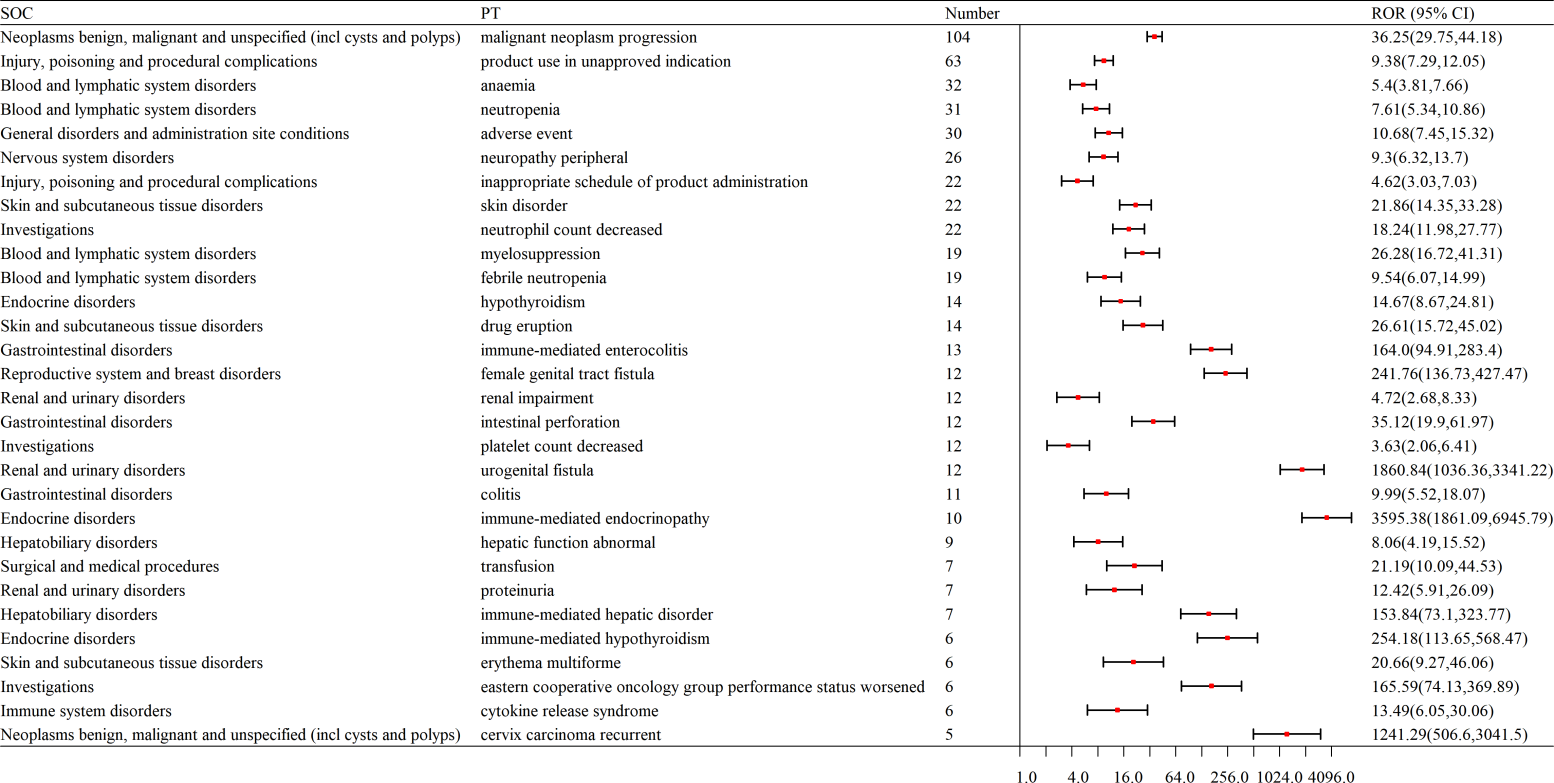
Forest plot of signal frequencies for AEs at the SOC level in the treatment of cervical cancer with pembrolizumab. SOC, System Organ Class; PT, Preferred Terms; ROR, Reporting Odds Ratio.

The top 30 signal frequencies for different system diseases are as follows. Hematologic disorders include anemia, neutropenia, febrile neutropenia, myelosuppression, and lymphadenopathy; neurological disorders include peripheral neuropathy; endocrine disorders include hypothyroidism and immune-mediated hypothyroidism; skin and subcutaneous tissue disorders include drug eruption, skin disorder, and erythema multiforme; reproductive system and breast disorders include female genital tract fistula and urogenital fistula; gastrointestinal disorders include immune-mediated enterocolitis, colitis, and intestinal perforation; renal and urinary disorders include renal impairment, proteinuria, and pyelonephritis; hepatobiliary disorders include abnormal hepatic function and immune-mediated hepatic disorder (as shown in **Figure 4**).

### 3.4 Distribution of AE signal intensity

**Figure 5:**
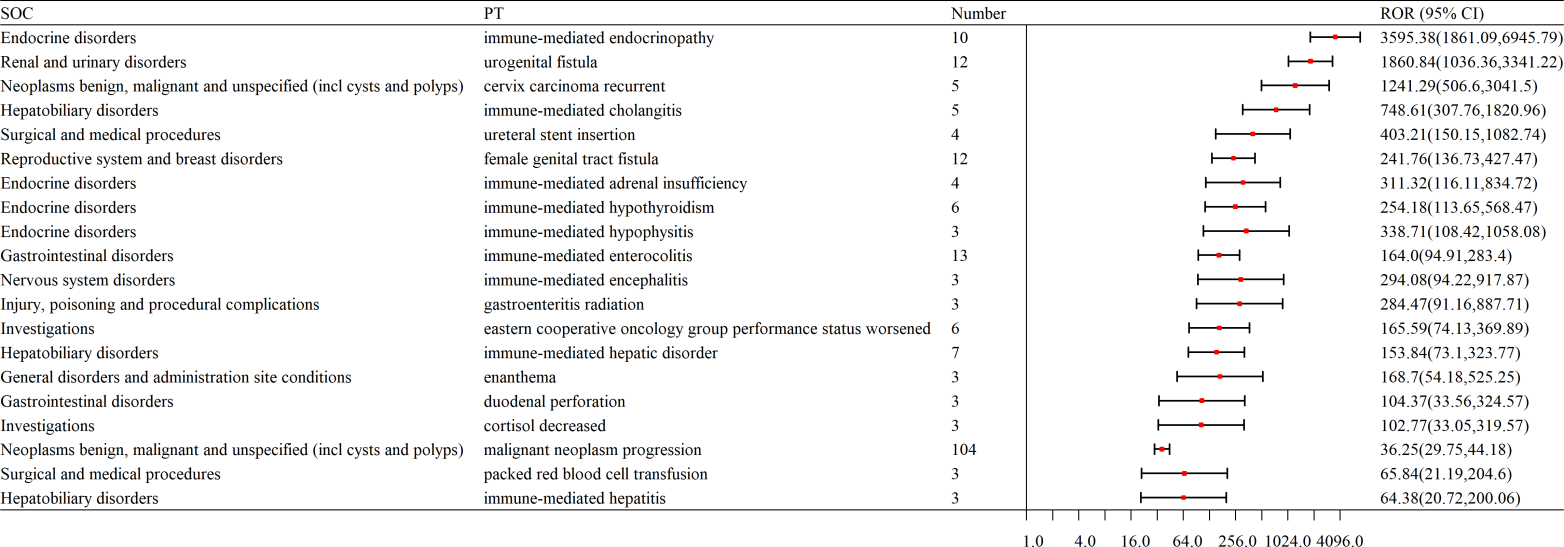
Forest plot of AE signal intensity at the SOC level in the application of pembrolizumab in cervical cancer. SOC, System Organ Class; PT, Preferred Terms; ROR, Reporting Odds Ratio.

The top 20 adverse events (AEs) ranked by signal strength distribution included:

Here is the organized list of the terms you provided: Immune-mediated Endocrinopathy; Urogenital Fistula; Cervix Carcinoma Recurrent; Immune-mediated Cholangitis; Ureteral Stent Insertion; Female Genital Tract Fistula; Immune-mediated Adrenal Insufficiency; Immune-mediated Hypothyroidism; Immune-mediated Hypophysitis; Immune-mediated Enterocolitis; Immune-mediated Encephalitis; Gastroenteritis Radiation; Eastern Cooperative Oncology Group Performance Status Worsened; Immune-mediated Hepatic Disorder; Enanthema; Duodenal Perforation; Cortisol Decreased; Malignant Neoplasm Progression; Packed Red Blood Cell Transfusion; Immune-mediated Hepatitis. Notably, endocrine system and immune-mediated disorders cannot be overlooked (as shown in **Table 4**, **Figure 5**).

**Table 4:** Signal strength of AEs at the SOC level in pembrolizumab-treated cervical cancer patients.

Number	PT	N	ROR (95%CI)	PRR(X^2^)	IC025/IC-2SD	EBGM05
1	immune-mediated endocrinopathy	10	3595.38(1861.09,6945.79)	3576.6(31817.91)	2.53	1647.98
2	urogenital fistula	12	1860.84(1036.36,3341.22)	1849.18(20836.24)	2.86	968.11
3	cervix carcinoma recurrent	5	1241.29(506.6,3041.5)	1238.05(5926.99)	1.37	484.58
4	immune-mediated cholangitis	5	748.61(307.76,1820.96)	746.66(3629.77)	1.38	299.26
5	Ureteral stent insertion	4	403.21(150.15,1082.74)	402.37(1579.55)	1.0	147.79
6	female genital tract fistula	12	241.76(136.73,427.47)	240.25(2835.64)	2.82	134.77
7	immune-mediated adrenal insufficiency	4	311.32(116.11,834.72)	310.68(1221.62)	1.0	114.65
8	immune-mediated hypothyroidism	6	254.18(113.65,568.47)	253.39(1495.28)	1.68	112.32
9	immune-mediated hypophysitis	3	338.71(108.42,1058.08)	338.18(996.91)	0.53	107.01
10	immune-mediated enterocolitis	13	164.0(94.91,283.4)	162.9(2080.12)	2.92	93.74
11	immune-mediated encephalitis	3	294.08(94.22,917.87)	293.62(866.1)	0.53	93.13
12	gastroenteritis radiation	3	284.47(91.16,887.71)	284.02(837.87)	0.53	90.14
13	eastern cooperative oncology group performance status worsened	6	165.59(74.13,369.89)	165.07(972.95)	1.66	73.48
14	immune-mediated hepatic disorder	7	153.84(73.1,323.77)	153.28(1053.48)	1.91	72.45
15	enanthema	3	168.7(54.18,525.25)	168.43(496.43)	0.52	53.79
16	duodenal perforation	3	104.37(33.56,324.57)	104.21(305.56)	0.51	33.39
17	cortisol decreased	3	102.77(33.05,319.57)	102.61(300.8)	0.51	32.88
18	malignant neoplasm progression	104	36.25(29.75,44.18)	34.33(3367.13)	4.41	28.14
19	packed red blood cell transfusion	3	65.84(21.19,204.6)	65.74(190.84)	0.49	21.11
20	immune-mediated hepatitis	3	64.38(20.72,200.06)	64.28(186.49)	0.49	20.64

PT, Preferred Terms; ROR, Reporting Odds Ratio; PRR, Proportional Reporting Ratio; IC025/IC-2SD, Information Component Lower Limit; EBGM05, Empirical Bayes Geometric Mean 5th Percentile.

### 3.5 Multivariable logistic regression analysis

Multivariable logistic regression analyses were employed to determine the immune-induced adverse events with pembrolizumab under different confounding factors. Older people (≥65 years) had a higher risk of immune-induced adverse event. Meanwhile, weight in 50-100kg was the protective factor, which would decrease the risk of immune-induced adverse events. Furthermore, weight more than 100kg and off-label use were irrelevant to immune-induced adverse event(as shown in **Table 5**).

**Table 5:** Multivariate logistic regression models of immune-related adverse events.

Variable	Adjusted OR (95%CI)	*P*
Age(years)		
<65	1.00 (Reference)	
≥65	1.32 (1.10-1.51)	<0.01
Weight (kg)		
<50	1.00 (Reference)	
50-100	0.69 (0.52-0.89)	<0.01
>100	1.02 (0.91-1.45)	0.35
Off-label use		
Yes	1.00 (Reference)	
No	0.88 (0.69-1.14)	0.19

OR, odds ratio; CI, confidence interval.

## 4 Discussion

The use of ICIs in cancer treatment is currently on the rise, and the number of irAEs has been increasing exponentially each year. Over 60% of these immune-related adverse events are associated with ipilimumab, nivolumab, and pembrolizumab (23). Pembrolizumab is currently the most widely used drug in immunotherapy for cervical cancer (24). However, due to the relatively short duration of pembrolizumab use in cervical cancer and limited clinical data, the specific adverse reactions associated with its application in cervical cancer are not yet fully understood. Therefore, this study systematically investigated and analyzed common signals of AEs related to pembrolizumab in cervical cancer using the FAERS database, providing valuable insights into the clinical safety of pembrolizumab in cervical cancer treatment.

The application of ICIs in cancer treatment has expanded significantly, yet their associated irAEs remain a major concern. The findings reveal that immune-mediated endocrinopathies are particularly prominent in cervical cancer treatment. Additionally, immune-mediated cholangitis, adrenal insufficiency, hypophysitis, enterocolitis, encephalitis, and hepatic disorders (hepatitis) were also observed. These adverse events suggest that pembrolizumab may trigger extensive autoimmune responses, affecting multiple organ systems. Therefore, elucidating its underlying mechanisms, enhancing early detection, and optimizing management strategies are crucial for improving its clinical safety.

Thyroid dysfunction is the most common endocrine toxicity associated with ICIs, characterized by a dynamic pathological evolution. Clinical data indicate that hypothyroidism occurs significantly more frequently than hyperthyroidism (25). However, recent studies suggest thyrotoxicosis may serve as an early warning signal. A case report documented a 39-year-old patient with metastatic melanoma who developed sudden palpitations and irritability—symptoms of thyrotoxicosis—before the third cycle of pembrolizumab combined with chemotherapy (26), suggesting hyperthyroidism as a potential early biomarker of immune activation. Notably, the dynamic transition of thyroid function may have prognostic implications. A retrospective study found that pembrolizumab-induced hyperthyroidism correlated with significantly improved survival outcomes (HR=0.11, P=0.038) (27), indicating that thyroid immune microenvironment activation may reflect systemic antitumor immune response intensity. The underlying molecular mechanisms involve multifaceted immune dysregulation. First, PD-1/PD-L1 pathway inhibition disrupts peripheral immune tolerance, leading to aberrant CD8+ T-cell activation and thyroid infiltration (28). Second, Th1 cytokines (e.g., IFN-γ, TNF-α) mediate thyroid follicular cell apoptosis, triggering transient thyrotoxicosis, followed by Hashimoto-like pathology with elevated thyroid peroxidase antibodies (TPOAb), ultimately progressing to permanent hypothyroidism (29). These pathological features suggest that baseline fT3 levels, combined with dynamic monitoring of thyroid autoantibodies (TPOAb, TgAb), may improve risk stratification.

Pembrolizumab-related endocrine toxicity often involves multiple glands. At the pituitary level, it impairs function via dual mechanisms. Cytotoxic T lymphocytes directly attack thyrotropin (TSH)-secreting cells, causing central hypothyroidism (30). Concurrently, inflammatory cytokine storms induce fibrotic remodeling of pituitary tissue, as evidenced in the KEYNOTE phase III trials—among 32 cases of pembrolizumab -induced hypophysitis, 11 developed severe ACTH deficiency, while none occurred in the placebo group (31). Adrenal dysfunction follows a cascade effect: PD-1 blockade promotes the production of 21-hydroxylase autoantibodies, directly damaging adrenal cortical cells, while inadequate pituitary ACTH secretion leads to secondary adrenal insufficiency. This dual insult exhibited a dose-dependent pattern in a four-year follow-up of a phase II clinical trial (32).

This study also identified cases of immune-mediated colitis in cervical cancer patients following pembrolizumab treatment, with a lower incidence compared to CTLA-4 inhibitors. A case report described a 59-year-old woman with advanced NSCLC who developed severe, persistent colitis after pembrolizumab therapy, requiring prolonged infliximab treatment even after anti-PD-1 discontinuation (33). These findings suggest that this adverse reaction is attributable to PD-1 inhibitors rather than a drug-specific effect of pembrolizumab. T-cell overactivation compromises intestinal barrier integrity, triggering an inflammatory response (34). Additionally, gut microbiota dysregulation—particularly alterations in Firmicutes—may contribute to ICI-associated colitis. Pembrolizumab-associated immune hepatitis is rare but can result in severe liver dysfunction (35). A 65-year-old woman with advanced lung adenocarcinoma developed severe delayed hepatitis following four cycles of pembrolizumab (36), supporting this study’s findings. Notably, hepatitis can occur even after ICI discontinuation, highlighting the diagnostic utility of liver biopsy (37). Clinicians should consider liver biopsy to confirm immune-mediated hepatitis. Furthermore, this study identified cases of encephalitis in cervical cancer patients following pembrolizumab treatment, suggesting immune-mediated encephalitis. A case report described a patient who developed severe immune-mediated encephalitis after pembrolizumab administration, presenting with profound confusion and mutism (38). Although the incidence of encephalitis associated with PD-1/PD-L1 inhibitors (e.g., pembrolizumab) is lower than that of CTLA-4 inhibitors, its prognosis can be poor when it occurs. Studies indicate that some patients with immune-mediated encephalitis test positive for anti-Hu, anti-Ma2, anti-LGI1, anti-CASPR2, or anti-NMDAR antibodies, which may disrupt synaptic function by targeting neuronal surface antigens (39). Moving forward, a combination of systemic and compartment-selective immunosuppressants may be essential to enable ICI therapy in patients with autoimmune conditions while mitigating irAEs.

In this study, hematologic adverse reactions in cervical cancer patients receiving pembrolizumab treatment included anemia, neutropenia, thrombocytopenia, and bone marrow suppression. In terms of the hematologic system, the use of pembrolizumab leads to an over-activated immune system that attacks normal bone marrow cells, resulting in bone marrow suppression and causing issues such as anemia, neutropenia, and thrombocytopenia (17, 40). However, in KEYNOTE-811 trail we found that anemia rates were even higher in the placebo group than in pembrolizumab (41), so the association between this adverse event and pembrolizumab needs to be further validated. A clinical study observed a probability of bone marrow suppression as high as 48.8% when pembrolizumab was combined with albumin paclitaxel and apatinib (42). This may be attributed to the PD-1 inhibitor lifting the inhibitory state of T cells by blocking the PD-1/PD-L1 signaling pathway, potentially leading to abnormal activation of T cells that attack hematopoietic stem cells or bone marrow stromal cells expressing normal antigens. On the other hand, paclitaxel drugs inhibit cell division by interfering with microtubule function and are clearly toxic to rapidly proliferating hematopoietic progenitors, especially neutrophil lines (43). Although the incidence of hematologic adverse reactions caused by ICIs is low, their impact is severe, and early detection and intervention are essential to optimize the management of hematologic adverse events.

The most common gastrointestinal adverse reactions associated with ICI treatment are diarrhea and colitis (14). Mild diarrhea can be managed with oral anti-diarrheal medications and treatment to correct electrolyte imbalances. If diarrhea lasts for more than one week, consideration should be given to performing a colonoscopy to confirm the presence of colitis and exclude the possibility of infectious diarrhea (44). A 2020 report described a case of advanced cervical cancer in which diarrhea occurred during ICI combination therapy. Colonoscopy showed normal intestinal mucosa, but pathological examination revealed significant lymphocytic infiltration in the distal colon epithelium, strongly suggesting colitis (45). Therefore, when colonoscopy is normal, pathological examination should be performed when necessary to confirm the diagnosis. For patients with severe colitis, corticosteroid treatment may be considered, and immunotherapy should be discontinued if necessary (46). In this study, gastrointestinal adverse reactions caused by pembrolizumab included colitis, duodenal perforation, intestinal perforation, and gastrointestinal perforation. Gastrointestinal perforation is a rare but serious complication of immune checkpoint inhibitor therapy, often secondary to colitis or gastrointestinal ulcers, and requires emergency treatment (47). During pembrolizumab treatment, gastrointestinal symptoms such as abdominal pain, nausea, vomiting, or bloating should be closely monitored, and timely intervention should be implemented if severe symptoms occur.

irAEs caused by ICIs primarily include maculopapular rashes, pruritus, and lichenoid dermatitis, with vitiligo being less common (48). The results of this study show that in cervical cancer patients treated with pembrolizumab, the skin irCAEs included drug-induced rashes and erythema multiforme, but vitiligo was not observed. Most skin-related irAEs occur within 6 weeks of ICI treatment. For mild rashes, topical corticosteroids can be used, while for severe rashes, oral corticosteroids may be added. If necessary, immunotherapy should be discontinued (49). Renal toxicity induced by ICIs is a relatively rare complication, with an incidence rate reported to be between 5-25% according to previous studies (50). Renal toxicity caused by ICIs typically does not show obvious clinical symptoms, and urine may present with proteinuria (51). In cases of suspected renal toxicity, empirical steroid treatment can be considered. If renal function does not improve after receiving steroid treatment, a kidney biopsy may be considered (52). Previous studies suggest that in cases of immune-related renal toxicity, ICIs should be immediately discontinued, and immunosuppressive treatment should be initiated (53). In this study, we also observed renal damage in cervical cancer patients treated with pembrolizumab. Therefore, renal function and urine protein should be monitored during pembrolizumab treatment. Among the cases in this study, there were 9 cases of female genital tract fistulas and 4 cases of uterine bleeding. However, no studies have currently shown a relationship between pembrolizumab and female genital tract fistulas or uterine bleeding. Common complications of cervical cancer include female genital tract fistulas and uterine bleeding (54). Therefore, the occurrence of female genital tract fistulas and uterine bleeding in cervical cancer may not necessarily be related to the use of pembrolizumab. Further research can be conducted in future clinical practice to validate this.

Previous studies have shown that combination therapy with ICIs results in a higher incidence and earlier onset of irAEs compared to monotherapy, and the incidence of irAEs is also related to the type of ICIs used (55). In general, PD-1 and PD-L1 inhibitors are better tolerated than CTLA-4 inhibitors (56). A review study showed that in CTLA-4 inhibitor therapy, grade 3 or 4 irAEs account for 31% of all irAEs, while in PD-1 inhibitor therapy, the incidence of grade 3 or 4 irAEs is 10% (23). In the gastrointestinal system, diarrhea is the most common irAEs, with an incidence of about 35% for CTLA-4 inhibitors, 20% for PD-1 inhibitors, and around 40% for combination therapy (57). In the endocrine system, thyroid dysfunction is the most common irAEs, and PD-1 inhibitors are more likely to cause such adverse reactions compared to CTLA-4 inhibitors (58). Additionally, the incidence of type 1 diabetes associated with CTLA-4 inhibitors is lower than that of PD-1 inhibitors (59). In a review of 59 clinical trials, the incidence of neurological-related irAEs was 3.8% for CTLA-4 inhibitors, 6% for PD-1 inhibitors, and 12% for combination therapy (60). A meta-analysis showed that the most common irAEs with Ipilimumab are dermatological diseases, gastrointestinal, and renal toxicity, while the most common irAEs with Pembrolizumab are joint pain, pneumonia, and liver toxicity, with Nivolumab showing predominantly endocrine toxicity, and Atezolizumab most commonly causing hypothyroidism (61). Currently, Pembrolizumab is the most widely used immune checkpoint inhibitor in the treatment of cervical cancer, while Nivolumab, Atezolizumab, and Durvalumab have also shown promising efficacy in some clinical trials (23). This study provides an in-depth discussion of the adverse effects of Pembrolizumab in cervical cancer treatment, offering a theoretical basis for clinical management of immune therapy in cervical cancer. In the future, further experiments can explore the use of other ICIs in cervical cancer and perform comparative analysis to provide a stronger theoretical foundation for drug selection in cervical cancer immunotherapy.

However, this study has some limitations. The data in the FAERS database comes from voluntary reports, which may lead to reporting bias. Specifically, certain adverse events may be reported frequently, while some milder or less common adverse events may be overlooked or not reported. Additionally, reports in the FAERS database often lack detailed clinical information, such as the severity of the disease, comorbidities, and medication history, which makes it more difficult to comprehensively assess the safety of the drug. Although disproportionality analysis has certain value in evaluating signal strength, it cannot quantify risk or prove causality between adverse events and the targeted drug, which presents challenges in determining the relationship between the two. In the future, further research based on multi-center, large-sample clinical data to explore the mechanisms of pembrolizumab’s adverse reactions in cervical cancer, along with the development of relevant diagnostic and treatment protocols, will help improve its safety assessment and provide stronger support for clinical practice.

## 5 Conclusion

Pembrolizumab is currently a major drug for immunotherapy in cervical cancer. However, there is still a relative lack of safety studies on pembrolizumab in large real-world populations. Therefore, our research is of great significance in this field. Based on the FAERS database, we systematically analyzed the adverse events associated with pembrolizumab in cervical cancer patients and identified common adverse event signals, particularly immune-mediated adverse events. This study found high-risk signals such as hematologic disorders, endocrine dysfunction, skin toxicity, and female reproductive system complications. Our research helps provide more reliable safety data for clinicians, guiding the development of individualized treatment plans and risk management. In addition, the results of this study may facilitate the early identification and timely intervention of immune therapy-related adverse events, ultimately improving treatment outcomes and the quality of life for patients.

## Data Availability

The original contributions presented in the study are included in the article/supplementary material. Further inquiries can be directed to the corresponding authors.
